# Data on solubilization, identification, and thermal stability of human Presenilin-2

**DOI:** 10.1016/j.dib.2018.01.039

**Published:** 2018-02-03

**Authors:** Ge Yang, Kun Yu, Jan Kubicek, Jörg Labahn

**Affiliations:** a Centre for Structural Systems Biology (CSSB), CSSB-FZJ, Notkestr. 85, 22607 Hamburg, Germany; b Institute of Complex Systems-Structural Biochemistry (ICS-6), Forschungszentrum Jülich, Wilhelm-Johnen-Str., 52425 Jülich, Germany; c Cube Biotech GmbH, Alfred-Nobel-Str. 10, 40789 Monheim, Germany

## Abstract

The data presented here are related to the research article entitled “Expression, purification, and preliminary characterization of human presenilin-2" [1].

Human Presenilin-2 is the catalytic subunit of γ-secretase and a possible calcium leakage channel (Kimberly et al., 2000; Tu et al., 2006) [2], [3]. HisPS2 which was obtained by overexpression in *E. coli* strain C43 (DE3) was extracted by detergent solubilisation. The sample isolation efficiency by detergents and the protein identification by mass spectrometry and western blot are described.

This data article describes the near and far UV circular dichroism measurements and the data deconvolution in terms of secondary structure at 4 and 98 °C. Also, a refolding spectrum is presented.

The raw CD spectra used for deconvolution of the helix and stand segments and average length are deposited into Protein Circular Dichroism Data Bank with PCDDBid: CD0005962000 (4 °C far UV), CD0005963000 (98 °C far UV), CD0005964000 (back to 4 °C far UV) and CD0005965000 (4 °C near UV CD).

**Specifications Table**

**Table:** 

Subject area	Biochemistry, Biophysics
More specific subject area	Human membrane protein
Type of data	Table, figure
How data was acquired	Bruker Daltonic Ultraflex III TOF/TOF mass spectrometer (Bruker Corporation, Billerica, USA)
	Flex Analysis Version 3.0 (Bruker Daltonics, Build 92)
	Aviv CD 425 (Aviv biomedical, Lakewood NJ,USA)
Data format	Analyzed
Experimental factors	HisPS2 was obtained from overexpression in *E. coli* strain C43 (DE3).
Experimental features	Solubilization, identification and thermal stability of hisPS2-detergent complex.
Data source location	Hamburg, Germany
Data accessibility	Data is with this article except for the CD data which are available at: http://pcddb.cryst.bbk.ac.uk/search.php
	The CD spectra can be accessed by searching with the PCDDBid given above (no login required).

**Value of the data**

• Data on solubilization efficiencies of membrane proteins are rare. Benchmarking the different effects of detergents from various classes on solubilization of membrane proteins provides relevant data for sample preparation and extends the existing knowledgebase of membrane protein solubilization.
• Sample characterization by MS and western blot is required for comparison of biophysical data obtained from samples.
• Near and far UV CD data of PS2 characterize the state of the protein and will allow for comparison with data of activated PS2 to elucidate changes in secondary and tertiary structure relevant for the acquisition of enzymatic functionality.
• The presented CD data show that PS2 does not unfold upon heating but changes its structure from mainly α-helix to β-strand by irreversible transition.

## 1 Data

Presenilins are the catalytic subunits of γ-secretase which produces the neuro-toxic Aβ peptides relevant for Alzheimer Disease [2], [3].

This data contains the table of the solubilization efficiency of 13 detergents from 3 classes with various chain length and charge properties in extracting hisPS2 from *E.coli* membrane. The sample identity is shown by MS and western blot. The secondary structure of PS2 is obtained from far UV CD data and the tertiary structure fingerprint by near UV CD. The CD data after re-cooling to 4 °C allow assessing the reversibility of structural transitions.

The identified peptides are shown in bold red. Transmembrane segment are in bold blue and underlined.

## 2 Experimental design, materials and methods

### 2.1 Detergent solubilization

Cell lysis and membrane preparation is similar as described in [4]. To perform the detergent screening, equal amount of membrane suspensions (correspond to 0.1 g cell pellet) containing the expressed hisPS2 was extracted by 0.5 ml solubilization buffer containing the respective detergent (Table 1). The detergent efficiency of extraction was classified by comparing the solubilization of hisPS2 in supernatant (solubilized fraction) and pellet (detergent resistant fraction) with the suspension before centrifugation at 100,000×g for 1 h (Table 1) by western blot [1]. (Table 2).

**Table 1:** Detergents efficiency in extracting hisPS2.

**Detergents**	**Short name**	**Type**	**Chain length**	**CMC (% W/V)**	**Percentage for solubilisation (% W/V)**	**Efficiency**
***n*****-Octyl-β-D-glucopyranoside**	OG	N	8	0.53	2	n
***n*****-Nonyl-β-D-glucopyranoside**	NG	N	9	0.20	1	n
***n*****-Decyl-β-D-maltopyranoside**	DM	N	10	0.087	1	p
***n*****-Dodecyl-β-D-maltoside**	DDM	N	12	0.0087	1	p
**6-Cyclohexylhexyl β-D-maltoside**	Cy6	N	12	0.028	1	p
***N,N*****-Dimethyldodecylamine**	LDAO	Z	10	0.023	1	n
***N*****-oxide**						
**FOS-choline-12**	FC12	Z	12	0.047	1	c
**FOS-choline-14**	FC14	Z	14	0.0046	1	c
**FOS-choline-16**	FC16	Z	16	0.00053	1	c
**3-[(3-Cholamidopropyl) dimethylammonio]-1-propanesulfonate**	CHAPS	Z	–	0.49	2	m
**3-([3-Cholamidopropyl] dimethylammonio)-2-hydroxy-1-propanesulfonate**	CHAPSO	Z	–	0.50	2	n
**Hexadecyltrimethylammonium bromide**	CTAB	C	16	0.034	1	n
***N*****-Lauroylsarcosine sodium salt**	NLS	A	12	0.42	1	c

Detergents name, abbreviation, type, hydrophobic chain tail length, cmc in percentage and the experimental concentration are listed. A: anionic; C: cationic; N: nonionic; Z: zwitterionic.

Solubilization efficiency: c, complete; m, marginal; n, no; p, partial.

**Table 2:** Irreversible transition of helix into strand upon thermal unfolding.

	**4 °C**	**98 °C**	**Back to 4 °C**
**Helix segments per 100 residues**	4.6±0.3	2.6±0.2	3.0±0.2
**Strand segments per 100 residues**	2.3±0.1	5.1±0.3	5.0±0.1
**Ave helix length per segment**	11.3±0.3	8.7±0.5	9.3±0.9
**Ave strand length per segment**	4.6±0.2	5.7±1.0	5.5±0.9

Number of helix and strand segments per 100 amino acids as well as the average helix and strand length are from the deconvoluted data as described above. Shown are the mean±standard deviation from the deconvolution results with reference data set 4, 7 and SMP 180.

### 2.2 Protein identification

**Fig. 1:**
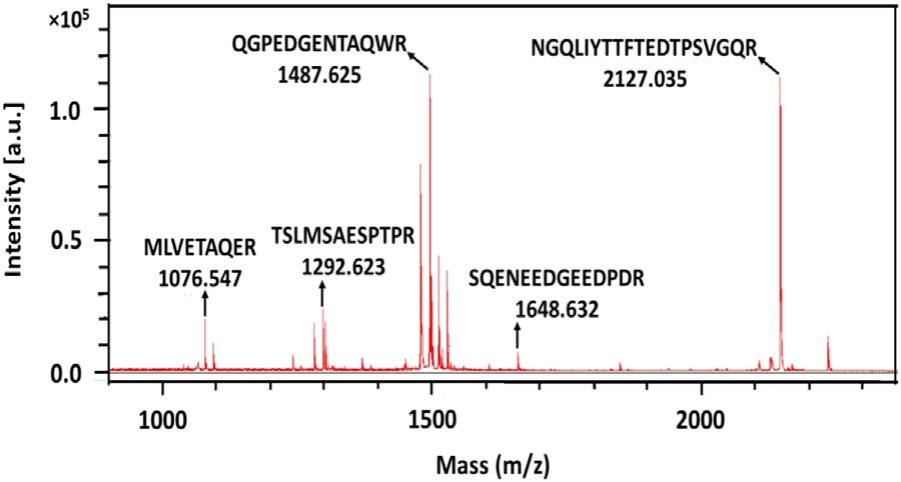
MALDI-TOF peptide mass fingerprint spectrum of hisPS2. Detected peptide sequences together with MW (Da) are indicated.

**Fig. 2:**
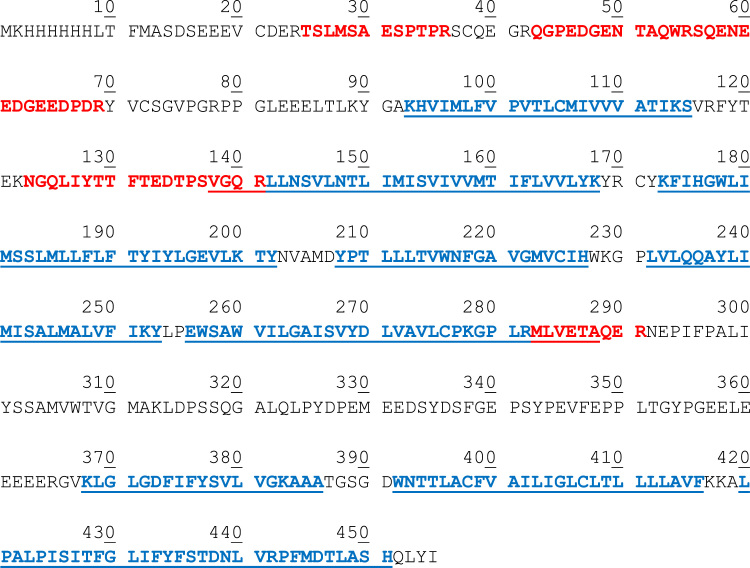
Location of the identified peptides by MS.

Chromatographically isolated trimeric full length hisPS2 [1] was analyzed on a 12% SDS-PAGE for his-tag positive western blot signals which were subjected to MS. Western positive bands were extracted and digested according to the Trypsin Profile IGD Kit (SIGMA) and acidified by addition of TFA to 0.1% final concentration. Sample of hisPS2 was analyzed by a Bruker Daltonic Ultraflex III TOF/TOF mass spectrometer using the software flex Analysis Version 3.0 (Bruker Daltonics, Build 92), and the peptides identified by searching against the protein database Swiss-Prot (Fig. 1, Fig. 2).

### 2.3 CD spectroscopy

The concentration of hisPS2 in CD buffer (10 mM sodium phosphate, pH 7.4, 0.014% (w/v) FC14) were determined by absorbance at 280 nm using the calculated extinction coefficient of 76780 M^−1^ cm^−1^ by Expasy ProtParam [5].

CD spectra were measured using an Aviv CD-425 spectrometer from 260 to 185 nm for far UV and 350 to 250 nm for near UV [1]. The far UV CD spectra were deconvoluted by CDSSTR using the reference data sets 4, 7 and SMP 180 [6].
