# Supplementary material for: Data on solubilization, identification, and thermal stability of human Presenilin-2
Source: Data Brief. 2018 Feb 3;17:626–30. doi: 10.1016/j.dib.2018.01.039 (PMC5852265; doi:10.1016/j.dib.2018.01.039)
Supplement: Supplementary file 1 — Supplementary material [file mmc1.docx]

There are no conflicts of interest.
